# Modulatory Role of Arbutin on Hepatic Inflammation and Apoptosis After Mild Repetitive Traumatic Brain Injury in Experimental Rats: Involvement of NGF/TrkA and IL‐6/JAK2/STAT3 Immune Signaling Crosstalk

**DOI:** 10.1155/bri/9048764

**Published:** 2026-07-13

**Authors:** Alaa S. Wahba, Amira A. El-Gazar, Dina M. Abo-Elmatty, Noha M. Mesbah, Mohamed A. Abdallah, Mohammed S. Sobh, Gehad M. Elnagar

**Affiliations:** ^1^ Department of Biochemistry, Faculty of Pharmacy, Suez Canal University, Ismailia, 41522, Egypt, scuegypt.edu.eg; ^2^ Department of Pharmacology and Toxicology, Faculty of Pharmacy, October 6 University, Sixth of October City, 12585, Egypt, o6u.edu.eg; ^3^ Biochemistry Department, Faculty of Pharmacy, El Saleheya El Gadida University, El Saleheya El Gadida, 44813, Egypt; ^4^ Department of Pathology, Faculty of Veterinary Medicine, Zagazig University, Zagazig, Egypt, zu.edu.eg; ^5^ Biochemistry Department, Faculty of Pharmacy, Zagazig University, Zagazig, Egypt, zu.edu.eg

**Keywords:** arbutin, hepatic dysfunction, mRTBI, neuroinflammation, NGF/TrKA, PI3K/AKt

## Abstract

Repetitive traumatic brain injury (RTBI) can cause long‐term complications, including persistent neuroinflammation, which can extend beyond the central nervous system, impacting various peripheral organs as liver. This study aimed to explore the neuroprotective and hepatoprotective effects of arbutin treatment in a rat model of mild RTBI (mRTBI), focusing on nerve growth factor (NGF)/tropomyosin receptor kinase A (TrkA) signaling pathway along with the crosstalk between brain injury and hepatic dysfunction. Animals were randomly assigned into three groups: one served as a normal control (NC) group, while the other two groups were exposed to one blow for 5 days and either left for one week after the fifth blow (mRTBI) or received arbutin intraperitoneally (100 mg/kg/day for 7 days, mRTBI + ARB). Biochemical and histopathological changes were monitored in the brain cortex and the liver. This study revealed that arbutin treatment offered neuroprotection and preserved most of the neuronal structures. Arbutin demonstrated a significant increase in the cortical NGF and TrkA contents, along with a marked upregulation in cortical phosphoinositol‐3 kinase (PI3K) and protein kinase B (AKt) mRNA levels compared to the mRTBI group. Furthermore, arbutin decreased cortical and serum inflammatory markers, reflecting its anti‐inflammatory power. Peripherally, arbutin treatment resulted in a substantial decrease in hepatic inflammatory markers, Janus kinase 2 (JAK2)/signal transducer and activator of transcription 3 (STAT3) and caspase‐3. These effects preserved hepatocellular histoarchitecture and reduced liver injury markers. Collectively, arbutin effectively modulated the NGF/TrkA signaling pathway, diminished inflammation, and alleviated the detrimental effects of mRTBI on both the brain and liver.

## 1. Introduction

Traumatic brain injury (TBI) is a major public health concern and one of the most prominent types of brain injuries. Moreover, it is the primary cause of disability and mortality among young individuals where it increases mortality, with a twofold higher death rate in the first year postinjury as compared to the general population [1].

Repetitive TBI (RTBI) occurs upon exposure to multiple TBI (often mild) where each successive injury may worsen the damage, leading to more severe long‐term effects. The latter can cause significant neurological decline, an increased risk of neurodegenerative diseases, cognitive decline, and emotional disturbance, with effects that are more severe and persistent than those of a single TBI [2].

The interaction between the brain and other organs adds another layer of complexity especially in pathological states. Previous studies have documented the reciprocal effects between an injured or diseased brain and various organ systems. For instance, TBI can negatively affect multiple organs, including the liver, lungs, gastrointestinal tract, cardiovascular system, kidneys, and immune system [3, 4]. The key mechanisms connecting TBI to peripheral organ dysfunction are the disruption of the blood–brain barrier, glial activation, cytokines production, autonomic dysregulation, and bidirectional organ dysfunction, whereas dysfunction in one organ potentiates injury to others [5].

Moreover, great attention to the crosstalk between the brain and liver, as one of the most sensitive peripheral organs to neuroinflammation, is highly required, where TBI can adversely affect liver function, primarily via the exaggerated systemic inflammation and cytokines signaling such as interleukin‐1β (IL‐1β), interleukin‐6 (IL‐6), and tumor necrosis factor‐alpha (TNF‐α) [6]. Liver, as a central organ in the immune response, reacts to increased circulating cytokines, producing additional inflammatory mediators and chemokines that recruit immune cells. This hepatic response amplifies the inflammatory cascade and can lead to liver impairment [7].

Arbutin, naturally derived from various plants like bearberry and blueberry, demonstrates numerous pharmacological effects including antimicrobial, disinfectant, anti‐hyperlipidemia, anti‐hyperglycemia, antioxidant, free radical scavenging, and antitumor and anti‐inflammatory activity [8]. Furthermore, previous studies revealed that arbutin exerted beneficial effects in supporting brain and liver health [9–11].

Therefore, the current study aimed to elucidate the crosstalk between repetitive head injury and liver dysfunction by tracking the underlying mechanism and signaling pathway in experimental rats. Another aim was to explore the possible protective mechanisms of arbutin against repetitive head injury and its effect on the liver.

## 2. Materials and Methods

### 2.1. Arbutin Source and Preparation

Arbutin (purity > 98%) was supplied from Acros Organics BV, a subsidiary of Thermo Fisher Scientific, located in Geel, Belgium. Arbutin was dissolved in distilled water and used freshly prepared.

### 2.2. Animals Care and Ethics Statement

Eighteen adults′ male Spraque‐Dawley (SD) rats, each weighing between 300 and 350 g, were obtained from the Holding Company for Biological Products & Vaccines SAE (VACSERA), located in Giza, Egypt. The rats were housed in stainless steel cages under environmentally controlled conditions including a 12‐h light/dark cycle, temperature (23 ± 2°C), humidity (60% ± 10%), and adequate ventilation. Prior to the initiation of experimental procedures, rats were allowed a minimum acclimatization period of 1 weeks and were permitted free access to food and water throughout the duration of the study.

Animal handling and experimental protocols were approved by the Suez Canal University Faculty of Pharmacy’s Ethics Committee (Ethics code: #202211PhDA4) and were conducted in accordance with the Guide for the Care and Use of Laboratory Animals (8^th^ edition, National Academies Press: Washington, DC, USA, 2011).

### 2.3. Mild RTBI (mRTBI) Induction

Trauma were induced adhering to our designed RTBI animal model [12]. Briefly, rats were placed on a platform directly under the weight drop device while being anesthetized with 4% isoflurane, maintained at a concentration of 1.5% through the experiment. Subsequently, 75 g weight with a sharp edge was dropped from a height of 25 cm, delivering an approximate impact energy of 0.5 J onto the exposed skull surface. The impact site was the right anterior frontal cortex, located 1.5 mm lateral to the midline at the midcoronal plane. This procedure was repeated once daily for five consecutive days to induce mRTBI.

### 2.4. Experimental Design

After the acclimatization period, three groups of rats were randomly assigned (*n* = 6/group). Group Ι served as the normal control (NC) group and was exposed to five repetitive days of 4% isoflurane, followed by 1.5% as a maintenance dose. Groups II and III were subjected to mRTBI consisting of one blow per day for five consecutive days. Group II was left untreated for 1 week after the final injury (mRTBI), while Group III received arbutin intraperitoneally at a dose of 100 mg/kg once daily for 7 days following the last injury (mRTBI + ARB) [13]. NC and mRTBI received IP injection of distilled water for 7 days, serving as a vehicle control.

### 2.5. Sample Collection and Processing

After the end of the experiment, rats were deeply anesthetized with thiopental sodium (100 mg/kg, i.p.) prior to blood collection. Blood samples were obtained from the femoral vein, after which euthanasia was completed. Sera were prepared from blood samples and kept at −20°C for subsequent analyses. After decapitation, rat brains were excised, and tissue samples were collected from the right anterior frontal cortex/peri‐impact site. These samples were divided into two portions: one portion was snap‐frozen in liquid nitrogen and stored at −80°C for biochemical and molecular analyses, whereas the other portion was fixed in 10% neutral buffered formalin for histological and immunohistochemical evaluation. Similarly, liver tissues were excised and rinsed in ice‐cold saline, and the left lateral lobe was divided into two portions, with one portion snap‐frozen at −80°C for biochemical and molecular analyses and the other fixed in 10% neutral buffered formalin for histopathological and immunohistochemical assessment.

### 2.6. Biochemical Parameters

Using colorimetric diagnostic kits (Spectrum, Cairo, Egypt), the serum levels of alanine aminotransferase (ALT) and aspartate aminotransferase (AST) were measured in accordance with the manufacturer’s instructions.

### 2.7. ELISA Determinations

Cortical contents of NGF, TrkA, and NF‐ҡB were measured in the brain tissue lysate using rat ELISA assay kits (NGF, Cusabio Biotech Co., Wuhan, CH; Cat. No. # CSB‐E04685r; TrkA and NF‐ҡB, MyBioSource; San Diego, USA; Cat. No. MBS8421511 and Cat. No. MBS453975, respectively), adopting the supplier’s protocols. Serum levels or homogenate contents of TNF‐α, IL‐1β, IL‐6, and IL‐10 were measured using ELISA kits (MyBioSource, San Diego, USA; Cat. No. MBS700574; Cat. No. MBS2023030; Cat. No. MBS2020158; Cat. No. MBS2020828, respectively) according to the manufacturer’s instructions.

Cortical and hepatic levels of JAK‐2 and STAT3 were analyzed using rat Elisa assay kit provided by MyBioSource, San Diego, USA (Cat. No. MBS2887358; Cat. No. MBS760293, respectively) adopting the supplier’s protocols.

### 2.8. Assessment of Gene Expression by Real‐Time PCR Analysis

RNA was extracted from cortical brain or hepatic tissue samples of the study groups using the ABT Total RNA Mini Extraction Kit (Applied Biotechnology, Ismailia, Egypt, Cat. No. ABT001), following the manufacturer’s instructions. The concentration and purity of the extracted RNA were determined using a NanoDrop 1000 spectrophotometer (NanoDrop Tech, Wilmington, DE, USA). The expression levels of phosphoinositol‐3 kinase (PI3K) and Akt genes were analyzed using the GoTaq 1‐Step RT‐qPCR System (Promega, Madison, WI, USA, Cat. No. A6020). Each reaction, with a final volume of 20 μL, consisted of a 4 μL RNA template, 0.4 μL GoScript RT mix for 1‐step RT‐qPCR, 1 μL of each forward and reverse primer, 10 μL GoTaq qPCR master mix, 0.31 μL additional CXR reference dye, and 3.29 μL nuclease‐free water.

The thermal cycling conditions were as follows: reverse transcription at 37°C for 15 min, enzyme inactivation at 95°C for 10 min, followed by 40 cycles of denaturation at 95°C for 10 s, annealing for 30 s, and extension at 72°C for 30 s. All real‐time PCR reactions were conducted using the StepOnePlus Real‐Time PCR thermal cycler (Applied Biosystems, Waltham, Massachusetts, USA). The mRNA expression levels of the examined genes were normalized to glyceraldehyde 3‐phosphate dehydrogenase (GAPDH) and represented as fold‐changes relative to the control group using the cycle threshold 2^−ΔΔCT^ [14]. The sequences of primers for the genes under investigation are detailed in Table 1.

**TABLE 1:** Primers sequence for RT‐PCR analysis.

Target gene	Primer sequence (5′‐3′)	Accession number
PI3K	F: AACACAGAAGACCAATACTC R: TTCGCCATCTACCACTAC	NM_013005.3

AKt	F: GTGGCAAGATGTGTATGAG R: CTGGCTGAGTAGGAGAAC	NM_033230.3

GAPDH	F: CCATCAACGACCCCTTCATT R: CACGACATACTCAGCACCAGC	NM_017008.4

### 2.9. Histological Assessment

Liver and brain samples were collected and preserved in 10% neutral buffered formalin for 24 h, dehydrated in graded ethanol, cleared in xylene, and embedded in paraffin. The tissue sections were cut into 5‐μm‐thick pieces, stained with hematoxylin and eosin (H&E), and viewed under a microscope to check for any histopathological changes [15]. A Swift microscope connected to a Swift digital camera (SW380T, USA) was used to take all the section photos. The histopathological lesions were estimated by semiquantitative methods as follows: “0 = absence of lesion, 1 = mild alterations, 2 = moderate alterations and 3 = severe alterations” [16].

### 2.10. Immunohistochemistry (IHC)

Rat liver and brain paraffin sections from various groups were stained using IHC in accordance with Hsu et al. [17] using rabbit polyclonal caspase‐3 antibody (#ab4051) at 1/200 dilution, rabbit polyclonal Bax antibody (#ab53154) at 1/50 dilution, rabbit polyclonal Bcl‐2 antibody (#ab59348) at 1/100 dilution for brain and liver tissues, and rabbit polyclonal TNF‐α antibody (#ab6671) at 1/200 dilution for brain tissues, Abcam, Cambridge, UK. The tissue sections from all experimental groups were dewaxed and hydrated. Staining was then performed using the DAB chromogenic agent (expose mouse and rabbit‐specific HRP/DAB detection kit, Abcam; ready‐to‐use; Cat. No. ab236466). Counterstaining by hematoxylin was done. All photos of the tissue sections stained by IHC were captured using a Swift microscope associated with Swift digital camera (SW380T, USA). Six representative areas total including both positive cell areas and areas devoid of expression were chosen for quantitative analysis, and an average value was determined for every animal.

### 2.11. Statistical Analysis

Prism 9 from GraphPad (San Diego, California, USA) was used to statistically analyze the results. Data were assessed for normality using the Shapiro–Wilk test. Parametric data are presented as mean ± standard deviation (SD) and were analyzed using one‐way analysis of variance (ANOVA) followed by Tukey’s post hoc test. Nonparametric data are presented as median (first to third quartiles) and were analyzed using the Kruskal–Wallis test followed by Dunn’s multiple comparisons test. The significance level was set at *p* < 0.05.

## 3. Results

### 3.1. Effect of Arbutin Post‐Treatment for 7 Days on Histopathological Changes in Brain Right Cortex After mRTBI Induction in Experimental Rats

**FIGURE 1:**
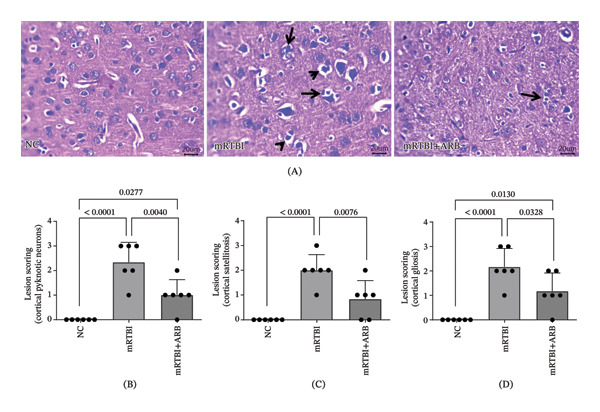
Effect of arbutin treatment for 7 days on mRTBI‐induced cortical histopathological changes (H&E stain; scale bar 20 μm). Representative cortical photomicrographs (A) show a normal neuronal architecture with intact neuronal cell bodies, neuropil, glial cells, and vasculature in the NC group. In contrast, the mRTBI group exhibits marked histopathological alterations, including numerous pyknotic neurons (arrowheads) and satellitosis (arrows). Arbutin treatment (mRTBI + ARB) markedly preserved cortical architecture, with restoration of neuronal morphology and near‐normal distribution of glial cells, although occasional degenerating neurons surrounded by glial cells were still observed (arrow). Magnification power: 400X. Histopathological lesion severity is presented for pyknotic neurons (B), satellitosis (C), and gliosis (D). Lesions were scored semiquantitatively as follows: 0 = absence of lesion, 1 = mild alterations, 2 = moderate alterations, and 3 = severe alterations. Data are expressed as mean ± SD and were analyzed using one‐way ANOVA followed by Tukey’s post hoc test (*n* = 6). *p* < 0.05 was considered significant. NC: normal control; mRTBI: mild repetitive traumatic brain injury; mRTBI + ARB: arbutin‐treated group.

Figure 1 displays the alterations in H&E‐stained sections from the right cortical tissues of the mRTBI group where numerous pyknotic neurons, satellitosis, neuronophagia, microgliosis, and dilated cerebral and meningeal vasculatures in contrast to the NC group, which showed normal histology of neuronal cell bodies, neuropil, glia cells, and vascular tissue. Semiquantitative analysis confirmed these observations, demonstrating a significant increase in lesion severity scores for pyknotic neurons, satellitosis, and gliosis in the mRTBI group compared with the NC group.

Notably, the arbutin treatment demonstrated an obvious neuroprotective effect, leading to the preservation of neuronal structure with few microgliosis detected in some fields. This improvement was further supported by a significant reduction in lesion severity scores for all assessed parameters compared with the mRTBI group. Arbutin treatment effectively mitigated reactive glial clustering (satellitosis), restoring it to near‐physiological levels.

### 3.2. Effect of Arbutin Post‐Treatment on the Cortical Contents of NGF and TrKA After mRTBI Induction in Experimental Rats

**FIGURE 2:**
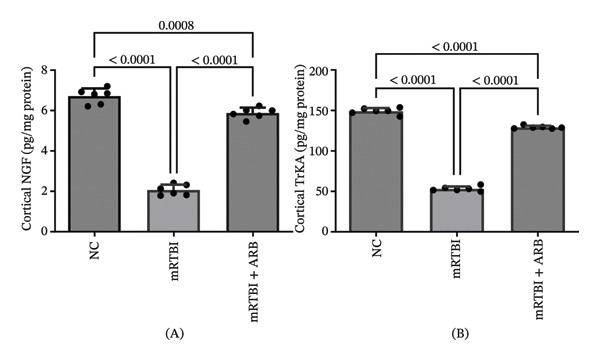
Effect of arbutin treatment for 7 days on the cortical contents of NGF (A) and TrKA (B) after mRTBI induction in rats. Data are expressed as mean ± (SD) and were analyzed using one‐way ANOVA followed by Tukey’s post hoc test; *n* = 6. *p* < 0.001 was considered significant. NC: normal control; mRTBI: mild repetitive traumatic brain injury; mRTBI + ARB: arbutin‐treated group; NGF: nerve growth factor, TrKA: tropomyosin receptor kinase A.

The cortical contents of NGF were significantly decreased in rats exposed to mRTBI by 69.25% [*F* (2, 15) = 385.6] as compared to NC. In contrast, arbutin post‐treatment for 7 days markedly increased NGF cortical contents by 2.85‐fold as compared to the mRTBI‐untreated group. NGF cortical levels in the arbutin‐treated group, although markedly restored, remained 12.49% lower than those of the NC group (Figure 2A).

Additionally, mRTBI rats displayed a significant decrease by 64.37% in the cortical content of TrkA [*F* (2, 15) = 1628] compared with healthy rat’s findings. Arbutin administration induced a 2.4‐fold increase in the TrKA cortical content compared with the traumatically injured rats. These findings indicated a marked restoration of TrkA, albeit remaining 13.35% lower than NC values (Figure 2B).

### 3.3. Effect of Arbutin Post‐Treatment on the Cortical mRNA Levels of PI3K and Akt as TrkA Signaling Genes After mRTBI Induction in Experimental Rats

**FIGURE 3:**
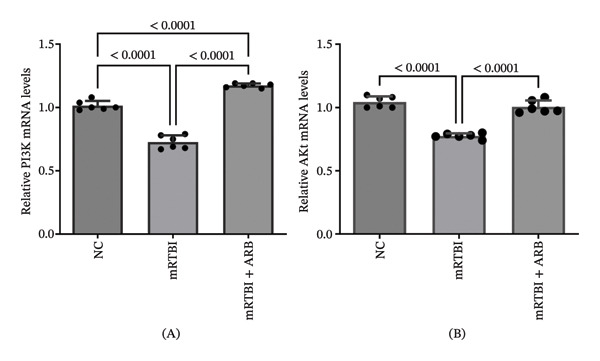
Effect of arbutin treatment for 7 days on the cortical mRNA levels of PI3K (A) and Akt (B) after mRTBI induction in rats. Data are expressed as mean ± (SD) and were analyzed using one‐way ANOVA followed by Tukey’s post hoc test; *n* = 6. *p* < 0.0001 was considered significant. NC: normal control; mRTBI: mild repetitive traumatic brain injury; mRTBI + ARB: arbutin‐treated group; PI3K: phosphoinositol‐3 kinase; Akt: protein kinase B.

Figure 3 shows a marked downregulation in cortical mRNA levels of PI3K by 28.4% [*F* (2, 15) = 204.8] and Akt by 25.7% [*F* (2, 15) = 76.21] in mRTBI rats, compared to the NC group. Arbutin treatment remarkably upregulated TrkA signaling gene expression by 1.6‐ and 1.3‐fold for PI3K and Akt, respectively, relevant to positive controls. Notably, arbutin treatment restored Akt mRNA expression to near‐normal levels (3.6% below NC) and significantly increased PI3K mRNA expression beyond normal levels by 15.57%, highlighting its enhancing effect.

### 3.4. Effect of Arbutin Post‐Treatment for 7 Days on the Cortical NF‐κB, IL‐1β, IL‐6, and TNF‐α Contents After mRTBI Induction in Experimental Rats

**FIGURE 4:**
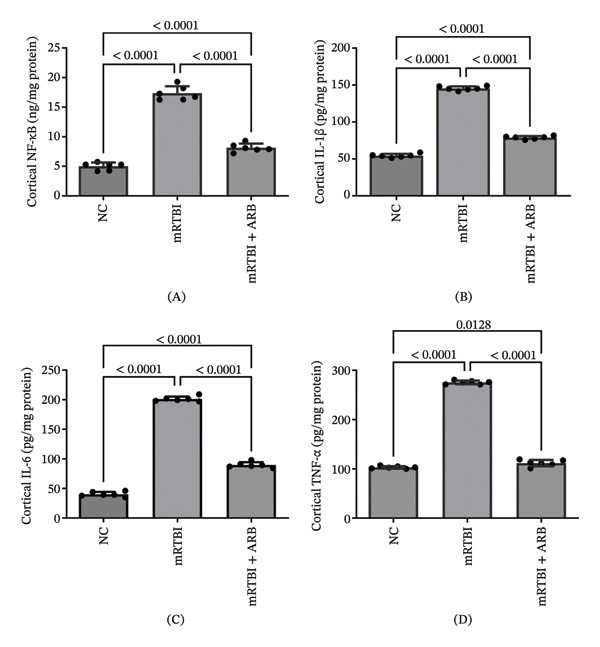
Effect of arbutin treatment for 7 days on the cortical contents of NF‐ҡB (A), IL‐1β (B), IL‐6 (C), and TNF‐α (D) after mRTBI induction in rats. Data are expressed as mean ± (SD) and were analyzed using one‐way ANOVA followed by Tukey’s post hoc test; *n* = 6. *p* < 0.05 was considered significant. NC: normal control group; mRTBI: mild repetitive traumatic brain injury; mRTBI + ARB: arbutin‐treated group; NF‐ҡB: nuclear factor kappa light‐chain enhancer of activated B cells; IL‐1β: interleukin‐1β; IL‐6: interleukin‐6; TNF‐α: tumor necrosis factor‐alpha.

As displayed in Figure 4, neuroinflammation was aggravated after the induction of mRTBI and was evident by the higher cortical contents of NF‐κB, IL‐1β, IL‐6, and TNF‐α in injured rats by 3.5‐, 2.67‐, 5‐, and 2.7‐fold, respectively, compared with NC ones. Meanwhile, arbutin treatment lowered the inflammatory response by 53.07% for NF‐ҡB [*F* (2, 15) = 317.5], 45.8% for IL‐1β [*F* (2, 15) = 2162], 55.4% for IL‐6 [*F* (2, 15) = 2134], and 59.4% for TNF‐α [*F* (2, 15) = 2734], compared to mRTBI. These results indicated that arbutin exhibited a potent anti‐inflammatory activity. Nevertheless, this reduction did not fully normalize inflammatory marker levels, which remained significantly higher than those of the NC group (62.66% for NF‐ҡB, 44.7% for IL‐1β, 123.3% for IL‐6, and 8.34% for TNF‐α).

### 3.5. Effect of Arbutin Post‐Treatment for 7 Days on Cortical Janus kinase 2 (JAK2) and STAT3 Signaling After mRTBI Induction in Experimental Rats

**FIGURE 5:**
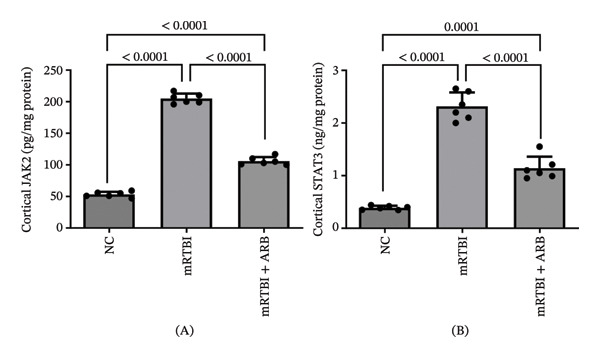
Effect of arbutin treatment for 7 days on the cortical contents of JAK2 (A) and STAT3 (B) after mRTBI induction in rats. Data are expressed as mean ± (SD) and were analyzed using one‐way ANOVA followed by Tukey’s post hoc test; *n* = 6. *p* < 0.0001 was considered significant. NC: normal control; mRTBI: mild repetitive traumatic brain injury; mRTBI + ARB: arbutin‐treated group; JAK2: Janus kinase2; STAT3: signal transducer and activator of transcription 3.

As illustrated in Figure 5, mRTBI induction markedly activated cortical JAK2/STAT3 signaling, as evidenced by a significant elevation in cortical JAK2 and STAT3 levels by 3.85‐ and 5.94‐fold, respectively, in injured rats compared with the NC group [*F* (2, 15) = 866.4 and 141, respectively].

Arbutin treatment significantly attenuated this activation, as evidenced by 48.2% and 50.71% decrease in JAK2 and STAT3 levels, respectively, relative to injured rats. These findings indicated that arbutin effectively suppresses the mRTBI‐induced activation of the JAK2/STAT3 signaling pathway. However, despite this marked attenuation, cortical JAK2 and STAT3 levels in the arbutin‐treated group remained elevated compared with the NC group, by approximately 99.36% for JAK2 and 192.97% for STAT3, indicating that arbutin partially, but not completely, restored normal signaling activity.

### 3.6. Effect of Arbutin Treatment for 7 Days on Cortical IHC Expression of TNF‐α After mRTBI Induction in Experimental Rats

**FIGURE 6:**
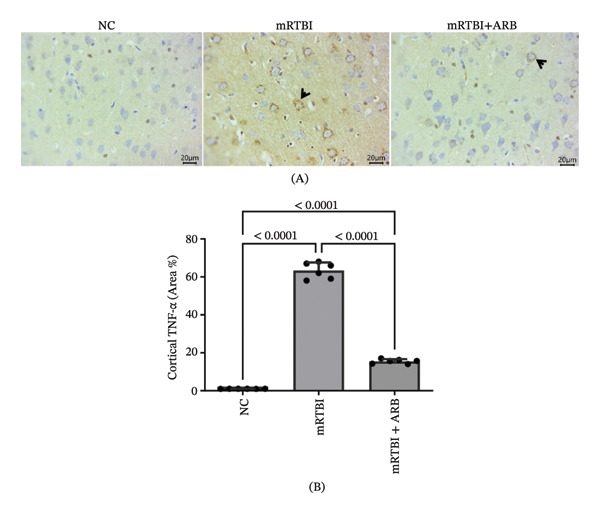
Effect of arbutin treatment for 7 days on the cortical TNF‐α immunoexpression (A and B) after mRTBI induction in rats. Representative photomicrographs of immunostained brain sections for TNF‐α (A) showed a nondetectable immunoexpression in neurons in the NC group, whereas in the mRTBI group, several numbers of positive immunostained cells were detected. Low numbers of labeled cells in the mRTBI + ARB group. IHC counterstaining with Mayer’s hematoxylin. Scale bar 20 μm. Arrowheads refer to the positively stained cells (the positively expressed cells revealed a golden brown color). Panel (B) depicted the area percentage of TNF‐α. Data expressed as mean ± (SD) and were analyzed using one‐way ANOVA followed by Tukey’s post hoc test; *n* = 6. *p* < 0.0001 was considered significant. NC: normal control; mRTBI: mild repetitive traumatic brain injury; mRTBI + ARB: arbutin‐treated group; TNF‐α: tumor necrosis factor‐alpha.

The mRTBI increased the cortical immunoexpression of TNF‐α (Figure 6A, B) by 60.4‐fold [*F* (2, 15) = 975.2] as compared to the healthy group that showed a nondetectable immunoexpression of TNF‐α. Arbutin administration showed the anti‐inflammatory effect marked by the significant decrease in the cortical immunoexpression of TNF‐α by 75.6% as compared to the mRTBI group. Although arbutin markedly ameliorated TNF‐α immunoexpression remained elevated relative to the NC group (by 14.7).

### 3.7. Effect of Arbutin Treatment for 7 Days on Cortical IHC Expression of Bcl‐2, Bax, and Caspase‐3 After mRTBI Induction in Experimental Rats

**FIGURE 7:**
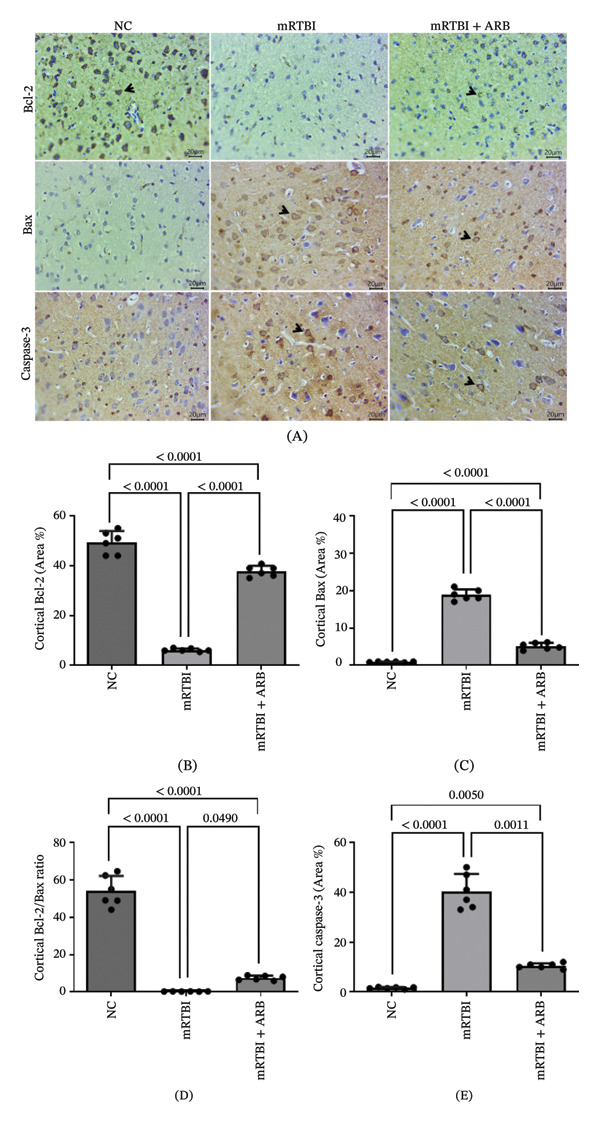
Effect of arbutin treatment for 7 days on the cortical immunoexpression of Bcl‐2, Bax, and caspase‐3 after mRTBI induction in rats. Representative photomicrographs of immunostained cerebral cortex sections (A) demonstrate a strong cytoplasmic expression of Bcl‐2 in numerous neurons in the NC group. In contrast, the mRTBI group exhibited a markedly reduced Bcl‐2 expression, whereas arbutin treatment (mRTBI + ARB) restored moderate cytoplasmic expression in neuronal cells. Bax immunoexpression was negligible in the NC group, while the mRTBI group showed an intense cytoplasmic expression in a large number of neurons. Arbutin treatment markedly reduced Bax expression, with only a few immunopositive cells observed. Similarly, caspase‐3 expression was absent in the NC group but showed a strong cytoplasmic expression in numerous neurons in the mRTBI group, indicating enhanced apoptotic activity. This expression was markedly attenuated following arbutin treatment, with only a limited number of immunopositive neurons detected. Immunohistochemical staining was counterstained with Mayer’s hematoxylin (scale bar = 20 μm). Arrowheads indicate the positively stained cells (brown coloration). Quantitative analysis of area percentage is presented for Bcl‐2 (B), Bax (C), and caspase‐3 (E), along with the calculated Bcl‐2/Bax ratio (D) as an index of apoptotic balance. Data are expressed as mean ± SD and were analyzed using one‐way ANOVA followed by Tukey’s post hoc test (*n* = 6). *p* < 0.05 was considered significant. NC: normal control; mRTBI: mild repetitive traumatic brain injury; mRTBI + ARB: arbutin‐treated group; Bcl‐2: B‐cell lymphoma 2; Bax: Bcl‐2–associated X protein.

Figure 7 showed a markedly reduced Bcl‐2 expression (Figure 7B) in the immunostained cerebral cortex sections of mRTBI by 87.43% [*F* (2, 15) = 343.2] as compared to the NC group, whereas arbutin treatment (mRTBI + ARB) restored moderate cytoplasmic expression in neuronal cells by 6.1‐fold compared with the mRTBI group. However, Bax immunoexpression (Figure 7C) showed an intense cytoplasmic expression in a large number of neurons by 20.39‐fold [*F* (2, 15) = 528.3] in the mRTBI group. Arbutin treatment markedly reduced Bax expression by 72.65% compared with mRTBI, with only a few immunopositive cells observed. As a result of a significant decrease of Bcl‐2 and increase of Bax immunoexpression, the Bcl‐2/Bax ratio (Figure 7D) was significantly decreased in the mRTBI group by 99.39% [*F* (2, 15) = 223.7] as compared to the NC group while increased following arbutin treatment by 22.65‐fold.

Additionally, caspase‐3 expression (Figure 7E) showed a strong cytoplasmic expression in numerous neurons in the mRTBI group by 24.8‐fold [*F* (2, 15) = 148.3] compared with NC group, indicating an enhanced apoptotic activity. This expression was markedly attenuated following arbutin treatment, with only a limited number of immunopositive neurons detected by 73.96% in comparison with the mRTBI group.

### 3.8. Effect of Arbutin Treatment for 7 Days on the Serum Levels of TNF‐α, IL‐1β, IL‐6, and IL‐10 After mRTBI Induction in Experimental Rats

**FIGURE 8:**
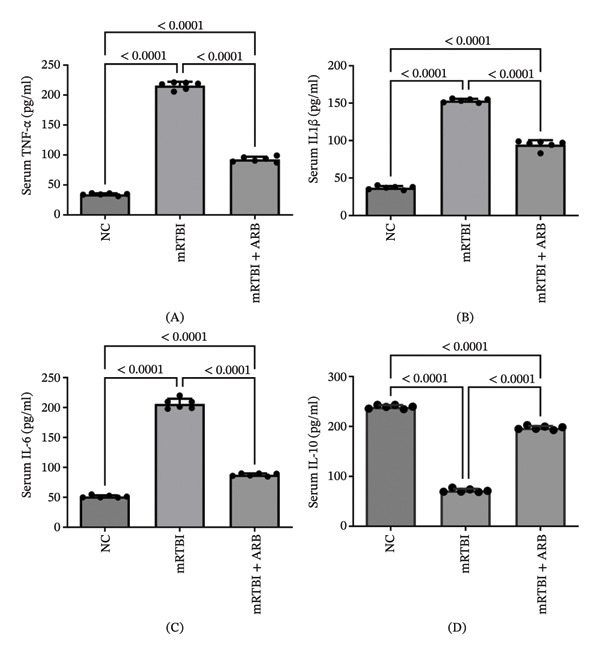
Effect of arbutin treatment for 7 days on the serum levels of TNF‐α (A), IL‐1β (B), IL‐6 (C), and IL‐10 (D) after mRTBI induction in rats. Data are expressed as mean ± (SD) and were analyzed using one‐way ANOVA followed by Tukey’s post hoc test; *n* = 6. *p* < 0.0001 was considered significant. NC: normal control; mRTBI: mild repetitive traumatic brain injury; mRTBI + ARB: arbutin‐treated group; TNF‐α: tumor necrosis factor‐alpha; IL‐6: interleukin‐6; IL‐1β: interleukin‐1β; IL‐10: interleukin‐10.

The positive control group displayed 6.3‐, 4.1‐, and 4‐folds elevations in serum levels of TNF‐α (Figure 8A), IL‐1β (Figure 8B), and IL‐6 (Figure 8C), respectively, compared to the negative control group. These increases were remarkably reduced by arbutin treatment by 57.1% [*F* (2, 15) = 2452], 38.3% [*F* (2, 15) = 1309], and 57.5% [*F* (2, 15) = 1458], respectively, compared to the mRTBI group. However, serum levels of TNF‐α, IL‐1β, and IL‐6 in the arbutin‐treated group remained higher than those of the NC group (by 169.56%, 155.62%, and 70.79%, respectively). On the other hand, serum levels of IL‐10 were decreased in mRTBI rats by 70.08% [*F* (2, 15) = 3501] as compared to the healthy control group. While arbutin administration significantly elevated serum IL‐10 levels by 2.75‐fold relative to injured rats, these levels remained 17.56% lower than those of the NC group (Figure 7D).

### 3.9. Effect of Arbutin Treatment for 7 Days on Serum Levels of ALT and AST as Liver Injury Markers After mRTBI Induction in Experimental Rats

**FIGURE 9:**
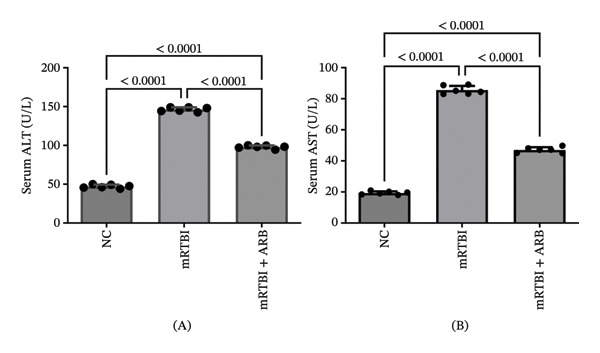
Effect of arbutin treatment for 7 days on the serum levels of ALT (A) and AST (B) after mRTBI induction in rats. Data are expressed as mean ± (SD) and were analyzed using one‐way ANOVA followed by Tukey’s post hoc test, *n* = 6. *p* < 0.0001 was considered significant. NC: normal control; mRTBI: mild repetitive traumatic brain injury; mRTBI + ARB: arbutin‐treated group; ALT: alanine aminotransferase; AST: aspartate aminotransferase.

As represented in Figure 9, rats exposed to mRTBI displayed significantly elevated serum levels of ALT and AST by 3.1 and 4.4 times, respectively, compared to normal rats. Treatment with arbutin for 7 days after repetitive trauma induction obviously reduced the elevation of ALT by 33% [*F* (2, 15) = 2571] and AST by 45.1% [*F* (2, 15) = 1720] compared to injured rats, reflecting the hepatoprotective effect of arbutin. Notably, although arbutin administration significantly attenuated the mRTBI‐induced elevations in ALT and AST, their serum levels remained higher than those of the NC group (by 108.11% and 143%, respectively).

### 3.10. Effect of Arbutin Post‐Treatment for 7 Days on Histopathological Changes in Hepatic Tissues After mRTBI Induction in Experimental Rats

**FIGURE 10:**
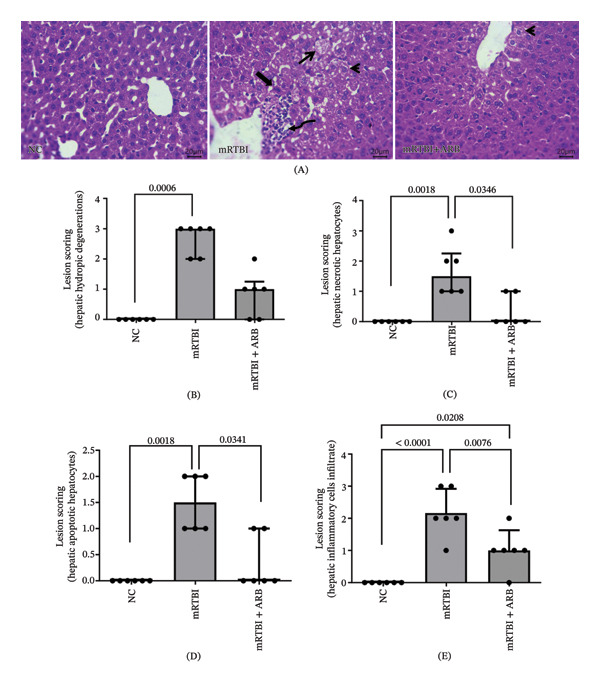
Effect of arbutin treatment for 7 days on mRTBI‐induced hepatic histopathological changes (H&E stain; scale bar 20 μm). Representative hepatic photomicrographs (A) showed a normal histology of hepatic cords and central vein in the NC group, while marked areas of hydropic degenerated hepatocytes (arrowhead), admixed with few numbers of apoptotic (thick arrow) and necrotic cells (thin arrow), in addition to the perivascular focal area of round cells (curved arrow) were seen in the mRTBI group. Mild degree of degenerative changes in some hepatocytes (arrowhead) and dilatation of sinusoids (thick arrow) was observed in the mRTBI + ARB group. Magnification power: 400X. Histopathological lesion severity is presented for hydropic degenerations (B), necrotic hepatocytes (C), apoptotic hepatocytes (D), and inflammatory cells infiltrate (E). Lesions were scored semiquantitatively as follows: 0 = absence of lesion, 1 = mild alterations, 2 = moderate alterations, and 3 = severe alterations. Data for hydropic degenerations, necrotic hepatocytes, and apoptotic hepatocytes are expressed as median (first–third quartiles) and were analyzed using the Kruskal–Wallis test followed by Dunn’s multiple comparisons test, whereas data for inflammatory cells infiltrate are expressed as mean ± SD and were analyzed using one‐way ANOVA followed by Tukey’s post hoc test (*n* = 6). *p* < 0.05 was considered significant. NC: normal control; mRTBI: mild repetitive traumatic brain injury; mRTBI + ARB: arbutin‐treated group.

Figure 10 represents the examined sections from hepatic tissue and its scoring where the NC group showed a normal histology of hepatic cords, Kupffer cells, sinusoids, portal triads, and central veins. The mRTBI group exhibited intense areas of hydropic degenerated hepatocytes and few numbers of necrotic cells and perivascular round cells infiltrates. Semiquantitative analysis confirmed these observations, demonstrating a significant increase in lesion severity scores for hydropic degeneration, necrosis, apoptosis, and inflammatory cell infiltration in the mRTBI group compared with the NC group.

Arbutin treatment markedly ameliorated these histopathological alterations, as evidenced by improved hepatic architecture and reduced degenerative changes. This improvement was further supported by a significant reduction in lesion severity scores for most assessed parameters compared with the mRTBI group. However, mild degenerative changes and slight sinusoidal dilation were still observed in some fields, indicating partial, but not complete, restoration of normal hepatic histology.

### 3.11. Effect of Arbutin Post‐Treatment for 7 Days on the Hepatic TNF‐α, IL‐1β, IL‐6, and IL‐10 Levels After mRTBI Induction in Experimental Rats

**FIGURE 11:**
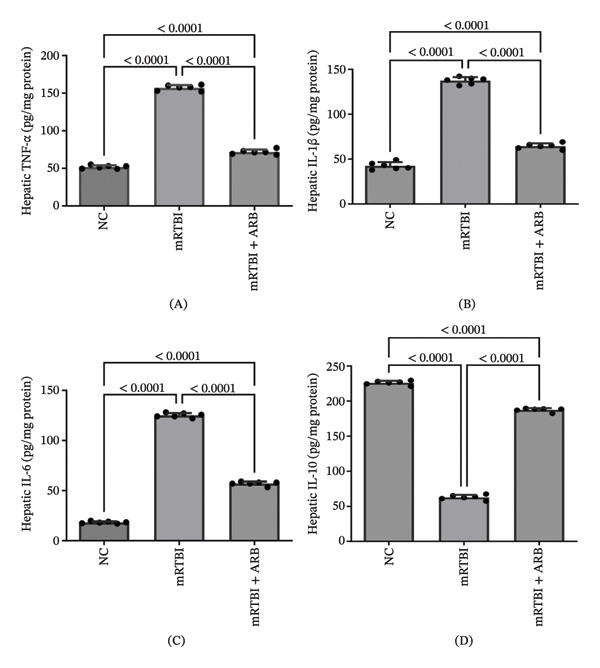
Effect of arbutin treatment for 7 days on the hepatic TNF‐α (A), IL‐6 (B), IL‐1β (C), and IL‐10 (D) after mRTBI induction in rats. Data are expressed as mean ± (SD) and were analyzed using one‐way ANOVA followed by Tukey’s post hoc test, *n* = 6. *p* < 0.0001 was considered significant. NC: normal control; mRTBI: mild repetitive traumatic brain injury; mRTBI + ARB: arbutin‐treated group; TNF‐α: tumor necrosis factor‐alpha; IL‐6: interleukin‐6; IL‐1β: interleukin‐1β; IL‐10: interleukin‐10.

Figure 11 demonstrated significant elevation in hepatic inflammatory mediators secondary to mRTBI induction including TNF‐α, IL‐1β, and IL‐6 by 3‐, 3.2‐, and 6.8‐fold, respectively, along with a decline in anti‐inflammatory IL‐10 by 72.2% as compared to the NC group. The treatment with arbutin confirmed its anti‐inflammatory power by significantly reducing the elevated levels of TNF‐α, IL‐1β, and IL‐6 by 54.34% [*F* (2, 15) = 1903], 53.2% [*F* (2, 15) = 1056], and 54.4% [*F* (2, 15) = 5288], respectively, while a significant increase in IL‐10 by 2.98‐fold [*F* (2, 15) = 4684] was demonstrated compared with positive controls data. This improvement, while substantial, did not achieve complete normalization of hepatic cytokine levels compared with NC values (TNF‐α (+38.4%), IL‐1β (+51.8%), IL‐6 (+211.98%), and IL‐10 (−82.96%) in comparison with the NC group.

### 3.12. Effect of Arbutin Post‐Treatment for 7 Days on Hepatic PI3K and Akt Signaling After mRTBI Induction in Experimental Rats

**FIGURE 12:**
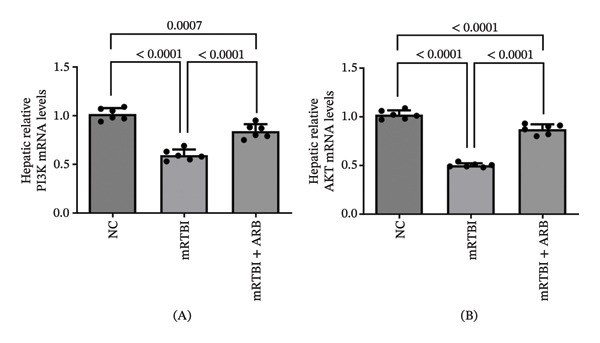
Effect of arbutin treatment for 7 days on the hepatic mRNA levels of PI3K (A) and Akt (B) after mRTBI induction in rats. Data expressed as mean ± (SD) and were analyzed using one‐way ANOVA followed by Tukey’s post hoc test; *n* = 6. *p* < 0.001 was considered significant. NC: normal control; mRTBI: mild repetitive traumatic brain injury; mRTBI + ARB: arbutin‐treated group; PI3K: phosphoinositol‐3 kinase; Akt: protein kinase B.

Given the close interplay between inflammatory mediators and intracellular survival signaling pathways, hepatic PI3K/Akt activation was further investigated to elucidate downstream molecular mechanisms. As illustrated in Figure 12, mRTBI induction resulted in a marked downregulation of hepatic PI3K and Akt mRNA expression compared with the NC group, corresponding to a 41.5% and 50.8% decrease, respectively [*F* (2, 15) = 64.79 and 245.3, respectively].

Arbutin treatment significantly restored hepatic PI3K and Akt expression levels compared with the mRTBI group, representing a 1.41‐ and 1.74‐fold elevation, respectively.

Despite this marked improvement, hepatic PI3K and Akt levels in the arbutin‐treated group remained slightly lower than those of the NC group, by approximately 17.4% for PI3K and 14.6% for Akt, indicating partial, but not complete, restoration of normal signaling activity.

### 3.13. Effect of Arbutin Post‐Treatment for 7 Days on the Hepatic Contents of JAK2 and STAT3 After mRTBI Induction in Experimental Rats

**FIGURE 13:**
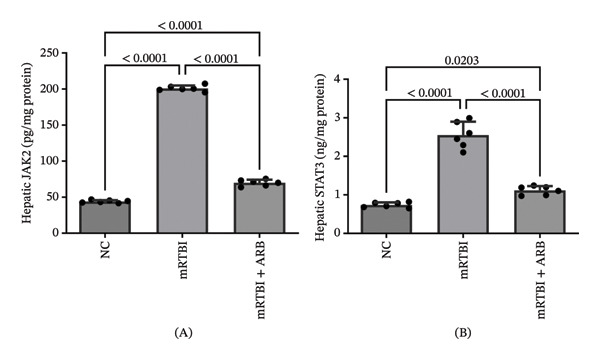
Effect of arbutin treatment for 7 days on the hepatic levels of JAK2 (A) and STAT3 (B) after mRTBI induction in rats. Data expressed as mean ± (SD) and were analyzed using one‐way ANOVA followed by Tukey’s post hoc test, *n* = 6. *p* < 0.05 was considered significant. NC: normal control; mRTBI: mild repetitive traumatic brain injury; mRTBI + ARB: arbutin‐treated group; JAK2: Janus kinase2; STAT3: signal transducer and activator of transcription 3.

As depicted in Figure 13, hepatic levels of JAK2 and STAT3 were significantly increased by 4.56‐ and 3.45‐folds relevant to control rats. Contrariwise, post‐treatment with arbutin significantly lowered these hepatic contents by 65.2% for JAK2 [*F* (2, 15) = 3260] and 56.3% for STAT3 [*F* (2, 15) = 121.4] in comparison with mRTBI. However, despite this marked reduction, hepatic levels of JAK2 and STAT3 in the arbutin‐treated group remained significantly higher than those of the NC group (by 59% and 51%, respectively).

### 3.14. Effect of Arbutin Post‐Treatment for 7 days on the Hepatic IHC Expression of Bcl‐2, Bax, and Caspase‐3 After mRTBI Induction in Experimental Rats

**FIGURE 14:**
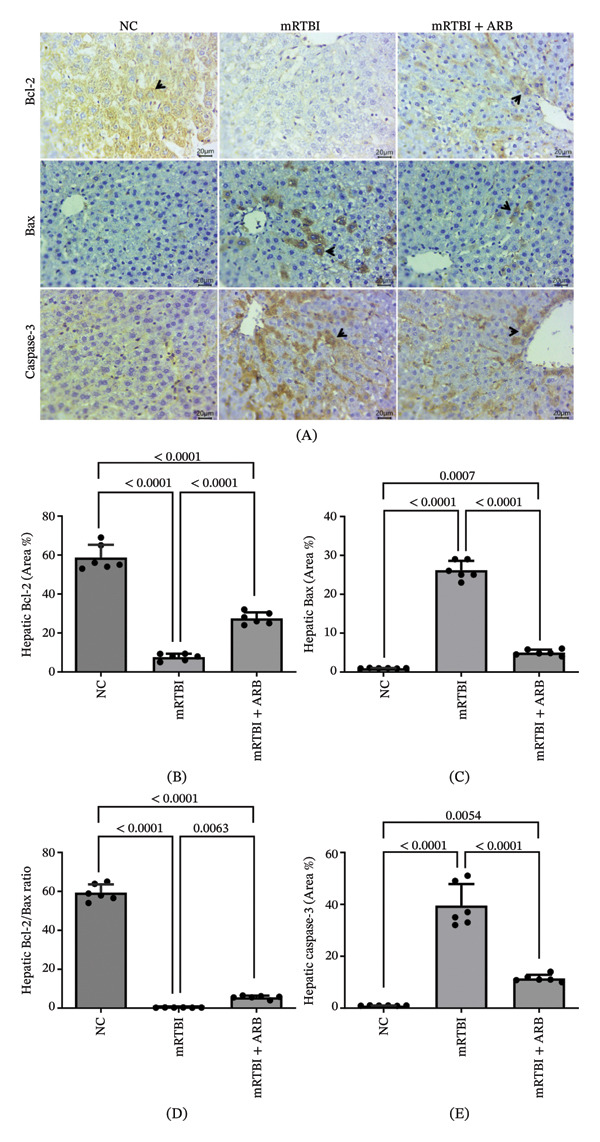
Effect of arbutin treatment for 7 days on the hepatic immunoexpression of Bcl‐2, Bax, and caspase‐3 after mRTBI induction in rats. Representative photomicrographs of immunostained liver sections (A) demonstrate a strong cytoplasmic expression of Bcl‐2 in numerous hepatocytes in the NC group. In contrast, the mRTBI group exhibited markedly attenuated Bcl‐2 expression, whereas arbutin treatment (mRTBI + ARB) restored moderate cytoplasmic Bcl‐2 expression in hepatocytes. Bax immunoexpression was negligible in the NC group, while the mRTBI group showed an intense cytoplasmic expression in a large number of hepatocytes. Arbutin treatment markedly reduced Bax expression, with only a few immunopositive cells observed. Caspase‐3 expression was absent in the NC group but was strongly expressed in the mRTBI group, indicating enhanced apoptotic activity. This expression was markedly reduced following arbutin treatment, with only a limited number of immunopositive hepatocytes detected. Immunohistochemical staining was counterstained with Mayer’s hematoxylin (scale bar = 20 μm). Arrowheads indicate the positively stained cells (brown coloration). Quantitative analysis of area percentage is presented for Bcl‐2 (B), Bax (C), and caspase‐3 (E), along with the calculated Bcl‐2/Bax ratio (D) as an index of apoptotic balance. Data are expressed as mean ± SD and were analyzed using one‐way ANOVA followed by Tukey’s post hoc test (*n* = 6). *p* < 0.01 was considered significant. NC: normal control; mRTBI: mild repetitive traumatic brain injury; mRTBI + ARB: arbutin‐treated group; Bcl‐2: B‐cell lymphoma 2; Bax: Bcl‐2–associated X protein.

Figure 14 demonstrates a marked attenuation in hepatic Bcl‐2 immunoexpression by 87.1% [*F* (2, 15) = 210.3], while an intense cytoplasmic expression in a large number of hepatocytes regarding Bax expression by 26.5‐fold [*F* (2, 15) = 518.4] was reported in the mRTBI group as compared to NC. On the other hand, a re‐establishment of cytoplasmic Bcl‐2 of hepatocytes was observed in the mRTBI + ARB group by a 3.6‐fold increase while markedly reduced Bax expression by 80.9% relevant to RTBI untreated one. As a result, the Bcl‐2/Bax ratio was significantly reduced by (99.5%, [*F* (2, 15) = 1012) compared to the NC group while increased in the mRTBI + ARB group by 19.3‐fold compared to the mRTBI group.

Moreover, an intense immune expression of caspase‐3 (40.4‐fold, [*F* (2, 15) = 99.93) in abundant hepatocytes in the mRTBI group compared with the NC group. However, there was a marked reduction in caspase‐3 immunoexpression by 70.96% in the mRTBI + ARB group.

## 4. Discussion

The connection between the dysfunction of extracranial organs and TBI is well recognized; thus, therapeutic strategies targeting the effects on extracranial organ systems after TBI hold substantial significance. Herein, we investigated for the first time to the best of our knowledge the ameliorative effects of arbutin on mRTBI‐induced liver inflammation and apoptosis in experimental rats via targeting NGF/TrkA/PI3K/AKt/NF‐ĸB and IL‐6/JAK2/STAT3 in the brain and IL‐6/JAK2/STAT3 and PI3K/AKt signaling in the liver using our established model of repetitive trauma in rats.

The mRTBI group exhibited significant neurodegenerative and inflammatory events, leading to both neuronal and hepatic dysfunctions, which may be attributed to the decreased NGF cortical contents. This observed reduction in NGF served as a critical initiating factor for a cascade of neuroinflammatory and pathological changes. The decrease in NGF likely impaired TrkA receptor signaling, disrupting neuroprotective pathways involving PI3K and AKt. This disruption was associated with increased levels of inflammatory cytokines (NF‐κB, TNF‐α, IL‐6, and IL‐1β) in the brain accompanied by the activation of the JAK2/STAT3 signaling pathway together with marked upregulation of Bax, decreased Bcl‐2 with caspase‐3 activation in the brain tissue, indicating a shift toward neuronal apoptosis in this tissue. Consequently, this inflammatory response extended to circulation and was associated with elevated hepatic proinflammatory cytokines, mainly IL‐6, and downstream activation of JAK2/STAT3 signaling alongside the downregulation of the PI3K/AKT survival pathway, as reflected by increased ALT and AST levels. An apoptotic profile, including Bax elevation, Bcl‐2 downregulation, and caspase‐3 activation, was observed in the brain and liver, highlighting a parallel pro‐apoptotic response in both organs and further underscored the liver’s response to the inflammatory milieu created by mRTBI. Thus, the decrease in NGF emerged as a pivotal factor that triggered a series of detrimental events following mRTBI, underscoring its importance in the pathophysiology of brain injury. Together, this study supports the link between repetitive trauma and liver dysfunction.

While some studies elicited an initial increase in NGF levels following TBI, as part of a neuroprotective response [18, 19], the long‐term effects of mRTBI on NGF levels remain vague. We aimed herein to elucidate the mechanisms underlying the observed decrease in NGF in our mRTBI rat model.

NGF, a member of the neurotrophin family, was first identified for its ability to promote growth, differentiation, survival, and the maintenance of different neurons [20]. It is particularly important due to its binding to the TrkA receptor, which activates various intracellular signaling pathways, notably the PI3K‐Akt pathway, which enhances neuronal survival by phosphorylating and inactivating various proteins [21, 22]. Another survival mechanism of NGF‐dependent signaling is inactivating NF‐ҡB [23].

The observed decrease of NGF in mRTBI in the current work may be attributed to several TBI effects as documented previously. These include mechanical stress on neuronal tissues that disrupts cellular functions and consequently NGF synthesis [24–27], or cellular metabolic pathways dysregulation resulting from TBI secondary injuries [28] that may impair the conversion of pro‐NGF to mature NGF, reducing its bioavailability and compromising the function of neuron [29–32]. This decline threatened the survival of NGF‐dependent neurons, leading to atrophy and increased neural damage, which created a vicious cycle exacerbating secondary brain damage following primary TBI insult.

All these were mirrored on histopathological findings in the mRTBI group, whereas rats exposed to only mRTBI revealed significant brain tissue damage, characterized by numerous pyknotic neurons, satellitosis, neuronophagia, microgliosis, and dilated cerebral and meningeal vasculature.

A significant inflammatory state was marked herein in the mRTBI group and signified by the increased cortical contents of NF‐ҡB, IL‐1β, IL‐6, and TNF‐α, which may have a role in the low cortical contents of NGF in the corresponding group. Augmenting our findings, the elevation in cytokine levels was reported to be correlated by the decreased NGF, by inhibiting its production or increasing its degradation that worsens neuronal health [33, 34]. Moreover, previous studies documented that NGF affect neuronal survival and modulate inflammatory responses via the TrkA receptor, activating the PI3K/AKt pathway via NGF‐induced Akt phosphorylation, which inhibited NF‐ҡB activation, thereby preventing inflammation and promoting neuronal plasticity [35–38]. Therefore, the reduced NGF observed in the mRTBI group likely impaired this protective pathway, hence increasing inflammation and neuronal damage, consistent with the elevated cytokine levels and histopathological findings in RTBI untreated group.

Moreover, as previously reported, active PI3K/AKt signaling was crucial for neuroprotection in experimentally induced parkinsonism by enhancing prosurvival factors and reducing neuroinflammation through the downregulation of NF‐ҡB [39]. In Alzheimer’s disease, neuroprotection was reported via NGF‐dependent TrkA activation [40, 41] while impaired NGF/TrkA signaling was implicated in its progression [42–46]. Concerning TBI, improvement in outcomes was observed with the intranasal NGF administration [28]. Collectively, these findings underscored the critical role of NGF and suggested that the reduced NGF in the mRTBI group may exacerbate secondary brain injury following the primary impact.

Conversely, the mRTBI + ARB group demonstrated the amelioration of histopathological changes of brain cortex, with a reduction in degenerative changes, while most neuronal structures were preserved, and the distribution of glial cells appeared normal. Furthermore, the presence of mild microgliosis in the mRTBI + ARB group may indicate an ongoing healing process in the brain. This aligns with the understanding that microgliosis is usually an indicator for healing process in the brain tissue [47]. Our findings farther demonstrated that arbutin treatment increased the cortical contents of NGF and TrkA along with higher PI3K and AKt mRNA cortical levels, coupled with a reduction in proinflammatory cytokines. To our knowledge, no existing studies have specifically examined the impact of arbutin on NGF levels in the context of mRTBI. Other studies have explored arbutin’s effects in other neurological conditions, such as parkinsonism, amyotrophic lateral sclerosis, multiple sclerosis, epilepsy, Huntington’s disease, and Alzheimer’s disease [10, 48–50]. Regarding arbutin anti‐inflammatory effect, numerous studies documented this potential formerly [51–53].

In the mRTBI + ARB group, arbutin treatment increased the PI3K/AKt cortical contents, a key downstream effector of NGF‐TrkA signaling [54]. This upregulation of PI3K/AKt not only promoted cell survival but also played a role in the anti‐inflammatory effects through decreasing the expression of proinflammatory cytokines (IL‐1β, TNF‐α, and IL‐6), by inhibiting the transcription factor NF‐ҡB. Additionally, it increased the anti‐inflammatory factor IL‐10, thereby reducing the inflammation and promoting an anti‐inflammatory environment [34, 51, 55–57]. By decreasing these inflammatory mediators, arbutin created a conducive environment for the elevation of NGF signaling in context with Furtado et al. [33].

The marked upregulation of JAK2/STAT3 in mRTBI brain tissue aligns with prior studies, confirming IL‐6/JAK2/STAT3 pathway activation post‐TBI [58, 59], driven by inflammatory signaling that enhances JAK2 phosphorylation, and STAT3 nuclear translocation [58, 60, 61]. This cascade was consistent with our NF‐κB and IL‐6 elevation in mRTBI [61, 62]. Additionally, JAK2/STAT3 activation culminates in increased Bax, decreased Bcl‐2, Bcl‐2/Bax imbalance, and caspase‐3 activation, matching reports of JAK2/STAT3‐mediated post‐TBI apoptotic events [59, 61].

Our findings support a sequential model in which neuroinflammation stimulates JAK2/STAT3 and Bax overexpression [61, 63], while parallel NGF/PI3K/AKT downregulation reduces Bcl‐2 and lowers the survival threshold, favoring caspase activation and neuronal loss [59, 61, 63, 64]. The treatment with ARB partially restored JAK2/STAT3 signaling toward homeostatic levels, consistent with its anti‐inflammatory profile and its established direct inhibition of JAK2/STAT3 phosphorylation in multiple inflammatory models [65–67], paralleling its suppression of IL‐6 production and cytokine release in LPS‐stimulated epithelial and immune cells via the modulation of the JAK2/STAT3 axis [53, 68], suggesting that it may effectively interrupting the IL‐6–JAK2–STAT3–NF‐κB amplification cycle in the context of mRTBI.

ARB concurrently blocks the proapoptotic JAK2/STAT3–Bax arm while enhancing the protective PI3K/AKT–Bcl‐2 pathway and increases the Bcl‐2/Bax ratio observed by immunohistochemical analyses and markedly suppressing caspase‐3 activity, consistent with prior evidence [54]. Collectively, these findings indicate that ARB reduces neuroinflammation, enhances NGF–PI3K/AKT signaling, and restores the Bcl‐2/Bax ratio, thereby inhibiting caspase‐3 activity and promoting neuroprotection, underscoring its role as a multitarget modulator of post‐mRTBI neuroinflammation and neuronal survival as evident herein by different biochemical and histopathological findings in the mRTBI + ARB group.

This study further investigated the secondary impact of trauma on the liver as an extracranial organ that is documented to be affected after TBI [69]. Our results revealed a significant hepatic alteration resulting from disruptions in the brain–liver axis following repetitive trauma. Whereas liver histopathological investigations showed marked areas of hydropic degeneration in hepatocytes, interspersed with a few numbers of necrotic cells, focal areas of round cells within the portal area, and dilated hepatic blood vessels. These alterations may be primarily attributed to systemic inflammatory responses and cytokine signaling observed after RTBI induction as the injured brain release proinflammatory cytokines such as TNF‐α, IL‐1β, and IL‐6, which enter the bloodstream and promote liver inflammation and damage as documented previously [6, 70, 71].

Activation of these pathways may cause further cytokine production and immune cell recruitment, amplifying inflammation in the liver, which is consistent with our findings, where a massive increase in systemic inflammation was associated with an elevation in hepatic proinflammatory cytokines such as TNF‐α, IL‐6, and IL‐1β and a reduction in anti‐inflammatory cytokine IL‐10. These findings aligned with the emphasis on neuroinflammation and its link with the peripheral system [72–74]. Additionally, these hepatic inflammatory changes were accompanied by a significant hepatic PI3K/AKt downregulation in the mRTBI group and in agreement with previous studies that reported IL‐6‐induced PI3K/AKt inhibition and inflammation‐suppressed PI3K/AKt hepatoprotection [75, 76].

Our findings also showed a massive increase in hepatic JAK2/STATЗ contents in the mRTBI group reflecting a systemic inflammatory response that may contribute to liver inflammation and dysfunction. This observation is in line with the findings of preclinical and clinical studies, which reported that the hepatocyte necrosis and liver injury are closely associated with the activation of the JAK2/STAT3 signaling pathway, which could be stimulated by cytokines such as TNF‐α and IL‐6 in response to brain injury [77, 78]. Additionally, our findings presented a disruption in liver homeostasis and hepatocyte damage, as indicated by the histopathological photomicrographs and increased serum levels of liver injury markers ALT and AST in the mRTBI group.

This disruption was further supported by studies examining how systemic factors such as inflammation could influence liver function in the context of brain injuries [79, 80]. The higher levels of ALT and AST observed in our study are consistent with other studies of TBI and its effect on liver function [81, 82]. Moreover, our results demonstrated an increase in hepatic apoptosis following mRTBI, signified by Bax and caspase‐3 upregulation, Bcl‐2 downregulation, and Bcl‐2/Bax ratio imbalance, consistent with previous studies showing that coordinated apoptotic alterations promote liver injury [83–87].

On the other hand, our findings suggests that ARB not only enhanced neurotrophins signaling and reduced neuroinflammation in the brain but also saved liver tissues, by reversing systemic inflammation and hepatic alterations associated with mRTBI. Where histoinvestigation revealed refinement in histological structures of hepatic parenchyma and vasculature in the mRTBI + ARB group, mild degenerative changes in some hepatocytes and dilated sinusoids were still observed in some examined fields.

ARB treatment significantly decreased liver injury markers ALT/AST along with reduced serum and hepatic proinflammatory cytokines TNF‐α, IL‐6, and IL‐1β, while increasing the anti‐inflammatory cytokine IL‐10, thereby supporting a hepatoprotective role for ARB in the liver, in addition to its primary anti‐inflammatory action [9, 88]. Furthermore, the ARB treatment resulted in a notable reduction in hepatic contents of the JAK2/STAT3, which further indicated a reduction in the inflammatory response [65]. This pathway is often linked to hepatocyte injury and inflammation as previously reported [78, 89, 90]. Simultaneously, ARB preserved PI3K/AKT pathway integrity through both inflammation suppression and direct pathway modulation, as evidenced by PI3K/AKT activation in arbutin‐treated models [54, 75, 91].

These improvements in JAK2/STAT3 inhibition and PI3K/AKt preservation restored hepatic Bcl‐2 levels, reduced caspase‐3 expression, and normalized the Bcl‐2/Bax ratio. This confirms comprehensive apoptosis reversal via established antiapoptotic mechanisms of both pathways [54, 67, 92, 93]. Hence, ARB fostered an anti‐inflammatory environment to liver as one of the most sensitive organs to inflammation. These findings align with previous studies showing that ARB alleviates liver injury by modulating apoptotic pathways to protect hepatocytes [9, 94].

## 5. Conclusion

**FIGURE 15:**
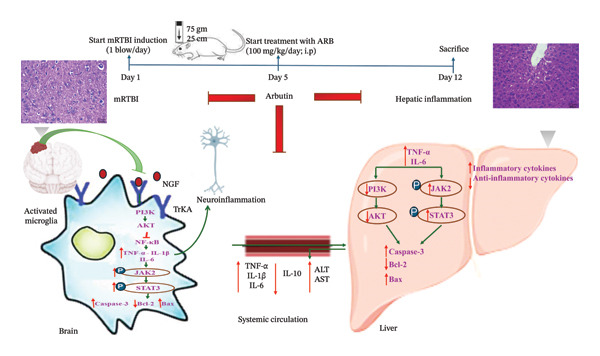
Graphical illustration of the neuroprotective and hepatoprotective effects of arbutin after mild repetitive traumatic brain injury induction.

The current study demonstrated significant neuroinflammatory and hepatic alterations, primarily linked to dysregulated NGF/TrKA pathway and downstream signaling, PI3K/AKt/NF‐ҡB and JAK2/STAT3 in RTBI model in rats. The mRTBI group exhibited increased proinflammatory cytokines and liver injury markers, indicating systemic inflammation. Conversely, arbutin treatment mitigated these effects by enhancing NGF signaling and reducing inflammation, leading to improved biochemical and histopathological outcomes in both brain and liver tissues via inhibiting hepatic JAK2/STAT3 and stimulating PI3K/AKt. This underscored arbutin’s potential as a therapeutic agent in managing mRTBI‐induced damage, highlighting the crosstalk of brain and liver responses to injury and inflammation. Further research is warranted to explore its clinical applicability in TBI management (Figure 15).

### 5.1. Study Limitations

The present study has certain limitations including the lack of a group given arbutin only and behavior tests. Future studies are recommended to address these limitations to further validate our findings.

## Author Contributions

Dina M. Abo‐Elmatty, Gehad M. Elnagar, Amira A. El‐Gazar, and Alaa S. Wahba: conceptualization and supervision. Mohamed A. Abdallah: methodology and investigation. Mohammed S. Sobh: methodology, visualization, and software. Gehad M. Elnagar, Amira A. El‐Gazar, and Alaa S. Wahba: methodology, formal analysis, data curation, and writing–original draft–review and editing. Dina M. Abo‐Elmatty and Noha M. Mesbah: writing–review and editing.

## Funding

This research did not receive any specific grant from funding agencies in the public, commercial, or not‐for‐profit sectors.

## Disclosure

The work was conducted as part of a PhD thesis at the Faculty of Pharmacy, Suez Canal University, Egypt. The study was carried out within the author’s institutional affiliations. The institutions had no role in the study design, data collection, analysis, manuscript preparation, or decision to publish.

## Conflicts of Interest

The authors declare no conflicts of interest.

## Data Availability

The data that support the findings of this study are available from the corresponding author upon reasonable request.
